# The Relation of Self-Esteem and Illegal Drug Usage in High School Students

**DOI:** 10.5812/ircmj.7682

**Published:** 2013-11-05

**Authors:** Mohammad Khajehdaluee, Abbas Zavar, Mahbobeh Alidoust, Razieh Pourandi

**Affiliations:** 1 Department of Social Medicine, School of Medicine, Mashhad University of Medical Sciences, Mashhad, IR Iran; 2 Addiction Research Center, Mashhad University of Medical Sciences, Mashhad, IR Iran; 3 Sarakhs Health Network, Mashhad University of Medical Sciences, Mashhad, IR Iran

**Keywords:** Self-Concept, Adolescent, Street Drugs

## Abstract

**Background:**

Adolescence is the period of stress and strain. Researchers have shown that adolescents without strong social supports would have tendency towards smoking and drug abuse.

**Objectives:**

This study aimed to evaluate the relationship between low self-esteem and illegal drug abuse.

**Materials and Methods:**

Participants were 943 grades nine to 12 high school students, from Sarakhs during 2010 - 2011. Adolescents participated in the study, completed two self-report questionnaires. The first questionnaire included questions about individual and family information, smoking and illegal drug abuse history, and the second was the Rosenberg's self-esteem scale.

**Results:**

53.8% of participants were male (507 individuals). The mean Rosenberg self-esteem score was 19.8 + 5.2, and the most frequent obtained scores were from 22 to 30. The difference of Rosenberg self-esteem score test between students who did not use any substance and those who had a history of smoking or drug abuse like heroin, pills, alcohols, betel nut (Nas) and other drugs (such as Pan and Hookah) was significant (P < 0.001). But this difference was not significant for marijuana (hashish) and opium. The difference of mean self-esteem scores between adolescents who lived with both or one of the parents, and those who did not live with any of parents, was significant (P = 0.04). There was also a significant association between the number of children in the family and self-esteem score.

**Conclusions:**

The current study showed significant association between the Rosenberg self-esteem test results and smoking, and illegal drug abuse like heroin, pills, alcohol, Nas, and other substances. Therefore, increasing self-esteem is essential for preventing the adolescents’ emotional and behavioral disorders. This fact could guide us to the new approaches for smoking and drug-abuse prevention in adolescents.

## 1. Background

Adolescence is the period of stress and strain. Researchers have shown that adolescents lacking strong social supports,would have tendency towards smoking and drug abuse (1). Recent researches have shown that drug abuse has been increased significantly among adolescents. The most important threat for this group is the possibility of facing repeated helplessness situations (such as feeling insecureness, pressure and emotional disturbances as well as having conflict with parents, friends or school), which may lead to seeking comfort in drugs (2-4). Based on the WHO reports, more than 100000 young people start using tobacco products and illegal substances each day, most of which live in the developing countries (5-8). Adolescents aged 11-19 constitute 27% of Iran population (16 million individuals) (9). Geographical, social, and cultural location of Iran, make it a suitable situation for adolescents and young people to lean towards drug addiction. There is limited information available about adolescents’ drug abuse in Iran. Countering against illegal drug trade and usage has been one of the most considerable efforts of Iranian government. Since addiction is a complex matter and legal prohibition methods are not helpful, in the recent years, attentions have been drawn to addiction prevention and treatment based on practical and scientific methods (10).

The most common description for self-esteem is the Rosenberg definition: a stable sense of personal worth or unworthy towards oneself. Feeling worthy influences all aspects of one’s life including the person’s view towards his or her abilities (11). Many students face experiences and problems which have never been occurred in their lives before. Students make efforts to reach their educational goals continuously and actively and are frequently evaluated by their teachers.. Furthermore, students are susceptible to psychological problems in different situations such as examinations, great deal of assignments, lack of leisure time, and longtime studying (12). Studying the Self-esteem concept is very valuable because researches have shown that the prevalence of behavioral and emotional disorders as well as antisocial behaviors (aggressiveness or violence, criminal activities, suicidal thoughts, smoking, abusing illegal drugs, school grades failing, irresponsibility, etc.) are more common among students with low self-esteem (13-17).

In the last few decades, efforts for reducing illegal drug abuse have drawn their attention from risk factors towards protecting factors (18). On the other hand, some studies have emphasized on the role of family besides personal properties including self-esteem as a protecting factor against illegal drug abuse (19). While some researchers reported a weak association between self-esteem and smoking or illegal drug abuse, some others reported strong association (20). Many studies have been performed on the relation between self-esteem and factors such as school progress, critical thinking, satisfaction, etc. (21), but there is a lack of evidence about the association between self-esteem and individual or social damages including illegal drug abuse.

## 2. Objectives

This study aimed to evaluate the relationship between low self-esteem and illegal drug abuse.

## 3. Materials and Methods

A cross sectional study was performed on high school and technical school students, grades nine to 12, from Sarakhs, Iran (41 schools with 5243 students) during 2010-2011. Sarakhs County is located in North East of Iran, neighboring Turkmenistan and Afghanistan. Ethnical diversity of this county is unique in Iran. This region is religiously [Shia (61%), and Sunni (39%)] and ethnically [33% Balochi, 33% Zaboli, 31% Persians, and 3% other ethnicities (Arabs, Turks and Kurds)] diverse. Calculated study size based on the estimation of proportion in population (α = 0.05 and d = 1.4p) was 325. The number was multiplied by three for cluster sampling (975). Thirty-two students were excluded from the study due to being absent in the day of filling the questionnaire, refusal of responding, and incomplete questionnaires. So the overall response rate was 96.7% with 975 participants and the final sampling number was 943. The study samples were divided into clusters with appropriate proportion to their population. In the next step, four schools were randomly selected from each cluster. The calculated study samples for each cluster were divided between schools appropriate to their population. In each school, students were selected with simple sampling method. They were justified by a team including researchers, consolers, teachers, and education experts. Two self-administrated questionnaires were completed by students. Totally, all data were considered confidential. Data were collected and recorded without names, just by code. The first questionnaire including demographic questions and questions about individual and family information, smoking and illegal drug abuse history (marijuana, opium, pills, alcohol, betel nut (Nas), and other substances) personally and in the family was filled. Any use of these substances was sufficient for that individual to be classified as being a drug user. No report was classified as never being a drug user.

We assessed self-esteem by the Persian version of the Rosenberg self-esteem questionnaire (3, 12, 20-22), ranging from 10 to 40, with lower scores indicating higher self-esteem. In the pilot study with 30 grade 10 students with a two-week interval, test-retest correlation was 0.79 (Cronbach’s alpha 0.83). The Rosenberg self-esteem scale is a self-esteem measurement widely used in social-science researches. It is a ten-item Likert-type scale with items answered on a four-point scale, from “strongly agree” to “strongly disagree”. Five of the items have positively worded statements and five have negatively worded ones. The scale measures state self-esteem by asking the respondents to reflect their current feelings. The Rosenberg self-esteem scale is considered a reliable and valid quantitative tool for self-esteem assessment. Statistical analysis was performed by SPSS 11.5 software, using analytical and descriptive statistical tests. Data were described with central indexes, distribution indexes and frequency. Chi-squared test was used to compare qualitative variables. To compare the self-esteem scores, Mann-Whitney, Kruskal-Wallis and Spearmen tests were used. P < 0.05 was considered significant in all of the calculations. The results were reported as Mean ± SD. Because of cluster sampling method, survey analysis was used in all the analyses. Ninety-five percent of the confidence interval was calculated for substance abuse prevalence. The Chi-square test, Fisher exact test, independent t-test, and logistic regression model were used for evaluating factors associated with substance abuse.

### 3.1. Ethical Considerations

Ethical approval was obtained from Mashhad University of Medical Sciences (MUMS) Research Ethics Committee. Permission was also received from MUMS for application of the data collection tools. Ethical issues (Including plagiarism, Informed Consent, misconduct, data fabrication and/or falsification, double publication and/or submission, redundancy, etc.) have been completely observed by the authors. The respondents were anonymous and participated willingly and voluntarily in this study. In addition, all of the responses were treated confidentially.

## 4. Results

Among all the questionnaires, 943 met the criteria to enter the study (3% missing). 55% were male, and the mean age of the students was 16.4 ± 1.1 years (min 14, max 19). Demographic and school information of participants are shown in Table 1. The mean of Rosenberg questionnaire self-esteem score was 19.8 ± 5.2 and the most frequent score was 22. Over 50% of students received a score more than 20 on the test. The mean of self-esteem score were 19.6 ± 5.2 in boys and 20 ± 5.2 in girls, but this difference was not significant by Mann-Whitney test (P = 0.25). Totally 227 (24%) students used illegal drugs, 62 students used alcohol, Betel nut (44), Opium (40), other substances (33) such as Pan and Hookah, Hashish (31), psychoactive pills (15) and Heroin (2) respectively. Table 2 demonstrated participants’ illegal drug abuse profiles and their relation with self-esteem score. The mean of self-esteem score in students who smoke, use heroin, alcohol and ecstasy pills was significantly lower than other students. The relation between self-esteem and using Nas and other substances (Pan and water pipe) was opposite; self-esteem was higher in this group of students. The mean of self-esteem score was reported lower in students who used marijuana and opium, but the difference was not significant. 

**Table 1.** Demographic, Family and School Characteristics of Participants

Variable	Male (n= 507, 55%)	Female (n = 436, 45%)	Total (n = 943)	P value
**School Grade**				0.58
9	157^a^ (31)	127 (29.1)	284 (30.1)	
10	165 (32.5)	157 (36)	322 (34.1)	
11	155 (30.6)	122 (28)	277 (29.4)	
12	30 (5.8)	30 (6.9)	60 (6.4)	
**School type**				< 0.001
Public	374 (73.8)	332 (76.1)	706 (74.9)	
Private	29 (5.7)	49 (11.2)	78 (8.3)	
School for talented	38 (7.5)	14 (3.2)	52 (5.5)	
Technical school	66 (13)	41 (9.4)	107 (11.3)	
**Field of study**				< 0.001
Sciences	53 (10.6)	32 (7.3)	85 (9.1)	
Mathematics	55 (11)	16 (3.7)	71 (7.6)	
Human Sciences	140 (27.9)	43(9.9)	183(19.5)	
General	138 (27.5)	117 (26.8)	255 (27.2)	
Others	115 (23)	228 (52.3)	343 (36.6)	
**Family situation (living with)**				0.98
Father and mother	456 (90.3)	391 (90.5)	847 (90.4)	
Only father	42 (0.8)	32 (0.7)	74 (0.7)	
Only mother	39 (7.7)	34 (7.9)	73 (7.8)	
Other relatives	6 (1.2)	4 (0.9)	10 (1.1)	
**Average school grade**				0.15
< 14	113 (22.5)	87 (20)	200 (21.3)	
14-18	345 (69.7)	304 (72.7)	649 (71.2)	
> 18	44 (8.8)	45 (10.3)	89 (9.5)	
**History of fail in a grade **	105 (20.8)	38 (8.7)	143 (15.2)	< 0.001
**Age, y**	16.39 ± 1.11^b^	16.34 ± 1.17	16.37 ± 1.14	0.53
**Father age**	47.13 ± 8.35	46.06 ±7.26	46.65 ± 7.91	0.06
**Mother age**	41.6 ± 7.08	40.68 ± 5.9	41.15 ± 6.72	0.05
**Number of children in the family**	3.62 ± 2.47	3.32 ± 2.28	3.48 ± 2.38	0.06
**Self-Esteem score**	19.61 ± 5.23	19.97 ± 5.24	19.79 ± 5.23	0.25

^a^ Frequency, %

^b^ Mean ± SD

**Table 2.** Mean of Self-Esteem Score Based on Illegal Drug Abuse

Substance	Frequency (%)	Self-Esteem Score	P value
**Smoking**			0.01
Yes	181 (19.2)	18.77 + 5.91	
No	762 (80.8)	20.04 +5.02	
**Marijuana (Hashish)**			0.81
Yes	31 (3.3)	20.1 +4.16	
No	912 (96.7)	19.77 + 5.27	
**Opium**			0.14
Yes	40 (4.2)	18.37 + 6.89	
No	903 (95.8)	19.85 +5.14	
**Heroin**			0.03
Yes	2 (0.2)	12.0 + 0	
No	941 (99.8)	19.8 +5.22	
**Pills**			< 0.001
Yes	15 (1.6)	14.92 +3.09	
No	928 (98.4)	19.86 +5.22	
**Alcohol**			< 0.001
Yes	62 (6.6)	17.51 +4.87	
No	881 (95.4)	19.96 + 5.22	
**Betel Nut (Nas)**			0.02
Yes	44(4.6)	17.86 + 5.97	
No	899 (95.4)	19.89 +5.17	
**Other substances**			0.04
Yes	33 (3.5)	17.97 +5.69	
No	910 (96.5)	19.85 +5.21	

The mean of Rosenberg questionnaire self-esteem score in students who lived with both parents was 19.8 + 5.22. The mean of self-esteem score for students who lived with their single father was 20.43 + 3.87, in those who lived with single mother was 19.97 + 4.9, and in students who lived with other relatives was 15 + 7.32. The differences of self-esteem scores between student groups based on people they lived with, were not significant. The differences of self-esteem scores between students who lived with other relatives and other groups (those who lived with one or both parents) were significant (P = 0.04). We found positive correlation between self-esteem scores with the number of family members, and also with the number of sisters (P = 0.02, r = 0.08). There was not any significant correlation between the number of brothers and self-esteem score. Some variables such as age, sex, history of grade failure, family situation (who they live with) could be considered as confounding factors in our study, but we did not find any association between them and self-esteem. There were 16 underlying causes of drug usage in abuser students, of which having fun, impact of peer and curiosity were the three most frequent ones. 68.7% of students were aware of drug usage disadvantages by Media (TV and radio), family and books respectively.

## 5. Discussion

The prevalence of substance abuse in this study was consistent with some previous national surveys (3, 4, 20, 23). In comparison to the other countries (24-27), the prevalence of substance abuse was considerably low in our study. Lower substance abuse rates among adolescents in Iran are mostly related to legal prohibition of illicit drugs, cultural values of Iranian families against substance abuse especially among adolescents, and strong parental disapproval of drug abuse. Based on our results, the prevalence of substance abuse in boys (53%) was much more than girls. Generally in the world, the rate of substance abuse and dependence is higher in men compared to women (28, 29). Moreover, based on the social norms and cultural values in Iran, the rates of substance abuse in women are much less than men (22).

There is a strong association between the adolescent age and substance abuse (30). In the present study, the prevalence of substance abuse was significantly increased by age of students and despite very low age variability and after controlling other variables, the risk of substance abuse was increased by increase of each year of age. Substance abuse in adolescents is a barrier to successful academic performance and academic achievement. Smokers as well as alcohol and other drug users, even those who have even used these substances once, tend to have worse scores and weaker school performances than other students (31). Our findings showed that the history of grade failure in substance abuser students was observed more than other students. Living with a single parent or in divorced families can make a teen more susceptible to drug abuse in a variety of ways. Substance useage in adolescents who live with a stepmother or stepfather is more than adolescents who live with both of their biological parents (32). Our results showed that the risk of substance abuse increased in students who do not live with both of their biological parents. Moreover, Fisher test found a significant association between self-esteem and poor family relationships in drug abusers (33).

Self-esteem is one of the human behavior stablishment factors. A person’s self-view and judgment determines his or her interactions with various issues. A person’s low self-esteem and self-respect, might lead to family and friends isolation as well as aggressive and antisocial behaviors. Self-esteem can be described as a collection of thoughts, feelings, emotions and experiences which are shaped through social life process. Considering thousands of personal experiences and self-assessments, a stable sense of worth or unworthiness gets built in the person (34). Adolescence is a period of life which connects childhood to adulthood. Changes in physical, mental, emotional, behavioral and cognitional aspects are very obvious in puberty. Struggles and contradictions are very common (35). Studies have shown that since unsuccessful students did not have sufficient successful experiences, they underestimated their own abilities and talents. The self-esteem in these type of students was very low (36). Students with high self-esteem are in better physical health and are successful in school (37, 38).

Our results showed a low prevalence of substance abuse in Iranian adolescents, and also determined some of its associated factors. By considering the high prevalence of addiction in the Iranian adult population (39), longitudinal studies on adolescent samples are suggested to determine the incidence rate of substance abuse and its related factors. The current study showed that the association between the Rosenberg self-esteem test results and smoking, and illegal abuses of drugs like heroin, pills, alcohol, Nas, and other substances was significant (the association was not significant for marijuana and opium abuse). Among native studies including Ayaytollahi et al. (20), Mohammadpor Asl et al. (3), and Mohammadpor Asl et al. (21), a significant association was also reported between the Rosenberg self-esteem test results and smoking. Other studies have reached similar conclusions in the world (40-44).

Increasing self-esteem is essential to prevent problems arising from low self-esteem in adolescents (45). Considering this fact would help to develop new strategies for preventing smoking and illegal drug abuse in adolescents. This means that while we are trying to prevent the substance abuse in adolescents with health educations and providing appropriate information, prevention by increasing their self-esteem might be more effective (20). High self-esteem is one of the strongest supports which can help students to face outdoor challenges. Therefore, families as well as school and society authorities have to keep the family, school and society environments in such a way that helps to increase students’ self-esteem. This may be possible through continuous and warm relationship between family members and their adolescent, reducing their emotional pressure, and improving their emotional and mental health (46). This way, family may help the teenager to develop appropriately and reach integrity. Presumably, holding off life skills courses, especially self-confidence, self-esteem increase and "say no" skill have significant impact on reducing illegal drug usage in teenagers and young adults. Simultaneously, organizing targeted programs for prevention and education of pupils, counseling centers for motivation increase, promoting positive mental attitudes and reducing risky behavior would be highly beneficial. The strength of present study was the sample selection from a multicultural zone (Sarakhs County), as representative of Iranian students.

This cross-sectional study had some limitations. First, the underlying causes such as psychosocial factors, smoking, alcohol, drugs access and friends and family attitudes were not fully investigated. Second, the limited number of schools, as well as ethical, social and legal limitations for assessment of sexual and other risk behaviors restricted the study. Fewer reported drug usage in such studies is common, because it is a social evil and may be a threat for the participants. In addition, the current study did not include students fired from school, in which the prevalence of substance abuse has been shown more than other students by numerous studies (47, 48). Therefore, the estimated prevalence of substance abuse reported above may represent lower estimates from the actual prevalence.

## References

[1] Kann L, Kinchen SA, Williams BI, Ross JG, Lowry R, Grunbaum JA (2000). Youth Risk Behavior Surveillance--United States, 1999. State and local YRBSS Coordinators.. J Sch Health..

[2] White VM, Hayman J (2006). Australian Secondary School Students' Use of Over-the-counter and Illicit Substances in 2005: Report..

[3] Mohammad Poorasl A, Vahidi R, Fakhari A, Rostami F, Dastghiri S (2007). Substance abuse in Iranian high school students.. Addict Behav..

[4] Momtazi S, Rawson R (2010). Substance abuse among Iranian high school students.. Curr Opin Psychiatry..

[5] Jha P, Chaloupka FJ (1999). Curbing the epidemic: governments and the economics of tobacco control. The World Bank.. Tob Control..

[6] Kessler DA (1995). Nicotine addiction in young people.. N Engl J Med..

[7] Saper CB (1995). Nicotine addiction in young people.. N Engl J Med..

[8] Ramezankhani A (1999). Study of the effect of application of the health credit pattern on preventive behaviors of conscripts smoking (dissertation)..

[9] Malekafzali H (2001). Fertility Health Public Education series..

[10] Hawkins JD, Catalano RF, Miller JY (1992). Risk and protective factors for alcohol and other drug problems in adolescence and early adulthood: implications for substance abuse prevention.. Psychol Bull..

[11] Moradi F, Ghanbari B, Aghamohammadian H (2011). The effectiveness of reality therapy in group method to enhance the self-esteem of the students of Ferdowsi university of Mashhad.. Stud Educ Psychol J..

[12] Moshki M, Ashtarian H (2010). Perceived health locus of control, self-esteem, and its relations to psychological well-being status in Iranian students.. Iran J Public Health..

[13] Remazani Disfani A (2003). Effectiveness of responsibility training with Glaser method on Identity crisis of high school students in Isfahan..

[14] Shihan I (2004). Self Esteem..

[15] Weinberg RS, Gould D (1999). Foundations of sport and exercise psychology.. J Human Kinetics..

[16] Guillon MS, Crocq MA, Bailey PE (2007). Nicotine dependence and self-esteem in adolescents with mental disorders.. Addict Behav..

[17] Ucman S, Prosen S (2007). Drug-addicted adults: Their self-concept, self-esteem and their role in treatment.. Eur Psychiat..

[18] Garmezy N (1993). Stressors of childhood..

[19] Mohammadi M, Jazayeri A, Rafiei H (2005). Family and individual variables in individuals at risk for substance abuse.. J Rehabil..

[20] Ayatollahi SA, Mohammadpoorasl A, Rajaeifard A (2005). Predicting the stages of smoking acquisition in the male students of Shiraz's high schools, 2003.. Nicotine Tob Res..

[21] Mohammadpourasl A, Fakhari A, Rostami F, Tabatabaei SM (2006). Multivariate Analysis of Psychological Factors Related to Adolescent Smoking.. Payesh J..

[22] Mohammadpoorasl A, Nedjat S, Fakhari A, Yazdani K, Foroushani AR, Fotouhi A (2012). Substance abuse in high school students in association with socio-demographic variables in northwest of iran.. Iran J Public Health..

[23] Ahmadi J, Hasani M (2003). Prevalence of substance use among Iranian high school students.. Addict Behav..

[24] Lampert T, Thamm M (2007). [Consumption of tobacco, alcohol and drugs among adolescents in Germany. Results of the German Health Interview and Examination Survey for Children and Adolescents (KiGGS)].. Bundesgesundheitsblatt Gesundheitsforschung Gesundheitsschutz..

[25] Ogel K, Corapcioglu A, Sir A, Tamar M, Tot S, Dogan O (2004). [Tobacco, alcohol and substance use prevalence among elementary and secondary school students in nine cities of Turkey].. Turk Psikiyatri Derg..

[26] Leatherdale ST, Ahmed R (2010). Alcohol, marijuana, and tobacco use among Canadian youth: do we need more multi-substance prevention programming?. J Prim Prev..

[27] Morvan Y, Rouvier J, Olie JP, Loo H, Krebs MO (2009). [Student's use of illicit drugs: a survey in a preventive health service].. Encephale..

[28] Brady KT, Randall CL (1999). Gender differences in substance use disorders.. Psychiatr Clin North Am..

[29] Khooshabi K, Ameneh-Forouzan S, Ghassabian A, Assari S (2010). Is there a gender difference in associates of adolescents' lifetime illicit drug use in Tehran, Iran?. Arch Med Sci..

[30] Smith BJ, Phongsavan P, Bauman AE, Havea D, Chey T (2007). Comparison of tobacco, alcohol and illegal drug usage among school students in three Pacific Island societies.. Drug Alcohol Depend..

[31] Engberg J, Morral AR (2006). Reducing substance use improves adolescents' school attendance.. Addiction..

[32] Coley RL, Votruba-Drzal E, Schindler HS (2008). Trajectories of parenting processes and adolescent substance use: reciprocal effects.. J Abnorm Child Psychol..

[33] Fisher LB, Miles IW, Austin SB, Camargo CA, Jr, Colditz GA (2007). Predictors of initiation of alcohol use among US adolescents: findings from a prospective cohort study.. Arch Pediatr Adolesc Med..

[34] Mackie MD, Smith RE (2002). Social psychology..

[35] Lotfabadi H (2009). Development Psychology: adolescence, young people and adults..

[36] Campbell JD, Fehr B (1990). Self-esteem and perceptions of conveyed impressions: is negative affectivity associated with greater realism?. J Pers Soc Psychol..

[37] Ruiz SY, Roosa MW, Gonzales NA (2002). Predictors of self-esteem for Mexican American and European American youths: a reexamination of the influence of parenting.. J Fam Psychol..

[38] Kermode S, MacLean D (2001). A study of the relationship between quality of life, health and self-esteem.. Aust J Adv Nurs..

[39] Sandeep C (2010). United Nations Office on Drugs Crime.

[40] Mouttapa M, Weiss JW, Hermann M (2009). Is image everything? The role of self-image in the relationship between family functioning and substance use among Hispanic adolescents.. Subst Use Misuse..

[41] Wills TA, Ainette MG, Stoolmiller M, Gibbons FX, Shinar O (2008). Good self-control as a buffering agent for adolescent substance use: an investigation in early adolescence with time-varying covariates.. Psychol Addict Behav..

[42] Barkin SL, Smith KS, DuRant RH (2002). Social skills and attitudes associated with substance use behaviors among young adolescents.. J Adolesc Health..

[43] Musher-Eizenman DR, Holub SC, Arnett M (2003). Attitude and peer influences on adolescent substance use: the moderating effect of age, sex, and substance.. J Drug Educ..

[44] Ruangkanchanasetr S, Plitponkarnpim A, Hetrakul P, Kongsakon R (2005). Youth risk behavior survey: Bangkok, Thailand.. J Adolesc Health..

[45] Teri R (1993). Self-esteem and self-efficacy of college students with disabilities.. Coll stud J..

[46] Amin Shokravi F, Memarian R, Hajizadeh E, Moshki M (2007). The roll of enhancing self-esteem educational program on self-esteem promotion of the girl students in Tehran Primary Schools.. Ofogh-e-Danesh J..

[47] Townsend L, Flisher AJ, King G (2007). A systematic review of the relationship between high school dropout and substance use.. Clin Child Fam Psychol Rev..

[48] Kogan SM, Luo Z, Brody GH, Murry VM (2005). The influence of high school dropout on substance use among African American youth.. J Ethn Subst Abuse..

